# Resin cement selection for different types of fixed partial coverage restorations: A narrative systematic review

**DOI:** 10.1002/cre2.761

**Published:** 2023-07-10

**Authors:** Safoura Ghodsi, Mina Shekarian, Mohammad Mostafa Aghamohseni, Sasan Rasaeipour, Sarah Arzani

**Affiliations:** ^1^ Dental Research Center, Dentistry Research Institute, Department of Prosthodontics Tehran University of Medical Sciences Tehran Iran; ^2^ Dental Research Center, Dental Research Institute, School of Dentistry Isfahan University of Medical Sciences Isfahan Iran; ^3^ Private Practitioner and Researcher Tehran Iran; ^4^ Fellowship in Implant Dentistry Tehran University of Medical Sciences Tehran Iran; ^5^ Child Growth and Development Research Center, Research Institute for Primordial Prevention of Non-Communicable Disease Isfahan University of Medical Sciences Isfahan Iran

**Keywords:** ceramics, dental bonding, dental veneer, resin cements, resin bonded ceramic

## Abstract

**Objective:**

The aim of this study was to review the selection criteria of resin cements for different types of partial coverage restorations (PCRs) and investigate if the type of restorations or restorative materials affect the type of selected resin cement.

**Materials and Methods:**

An electronic search (1991–2023) was performed in PubMed, Medline, Scopus, and Google Scholar databases by combinations of related keywords.

**Results:**

A total of 68 articles were included to review the selection criteria based on the advantages, disadvantages, indications, and performance of resin cements for different types of PCRs.

**Conclusions:**

The survival and success of PCRs are largely affected by appropriate cement selection. Self‐curing and dual‐curing resin cements have been recommended for the cementation of metallic PCRs. The PCRs fabricated from thin, translucent, and low‐strength ceramics could be adhesively bonded by light‐cure conventional resin cements. Self‐etching and self‐adhesive cements, especially dual‐cure types, are not generally indicated for laminate veneers.

## 1 INTRODUCTION

Recent advances in adhesive dentistry, parallel to the rise in esthetic demand, have increased the indications for partial coverage restorations (PCRs) (Morimoto et al., 2016; Thordrup et al., 2006). A PCR (namely, inlay, onlay, laminate veneer, endocrown, etc.) is a type of fixed restoration that does not cover the whole external tooth surface. This conservative indirect restoration restores the tooth's integrity while preserving the intact remaining tooth structure (Donovan & Chee, 1993).

The cementation procedure could be discussed in two different categories: conventional cementation and adhesive luting. Conventional cementation might be performed by different types of conventional (e.g., zinc phosphate, glass ionomer) or resin cements and mainly relies on mechanical bonding. Adhesive luting is accomplished by resin cements and benefits from a combination of mechanical, micromechanical, chemical, and molecular bonding mechanisms (Kameyama et al., 2015; Sakaguchi et al., 2019). The cementation of full‐coverage restorations could be done by both of these methods; however, PCRs call for adhesive cementation for esthetic, strength, and durable retention (Gresnigt et al., 2021).

**Figure 1:**
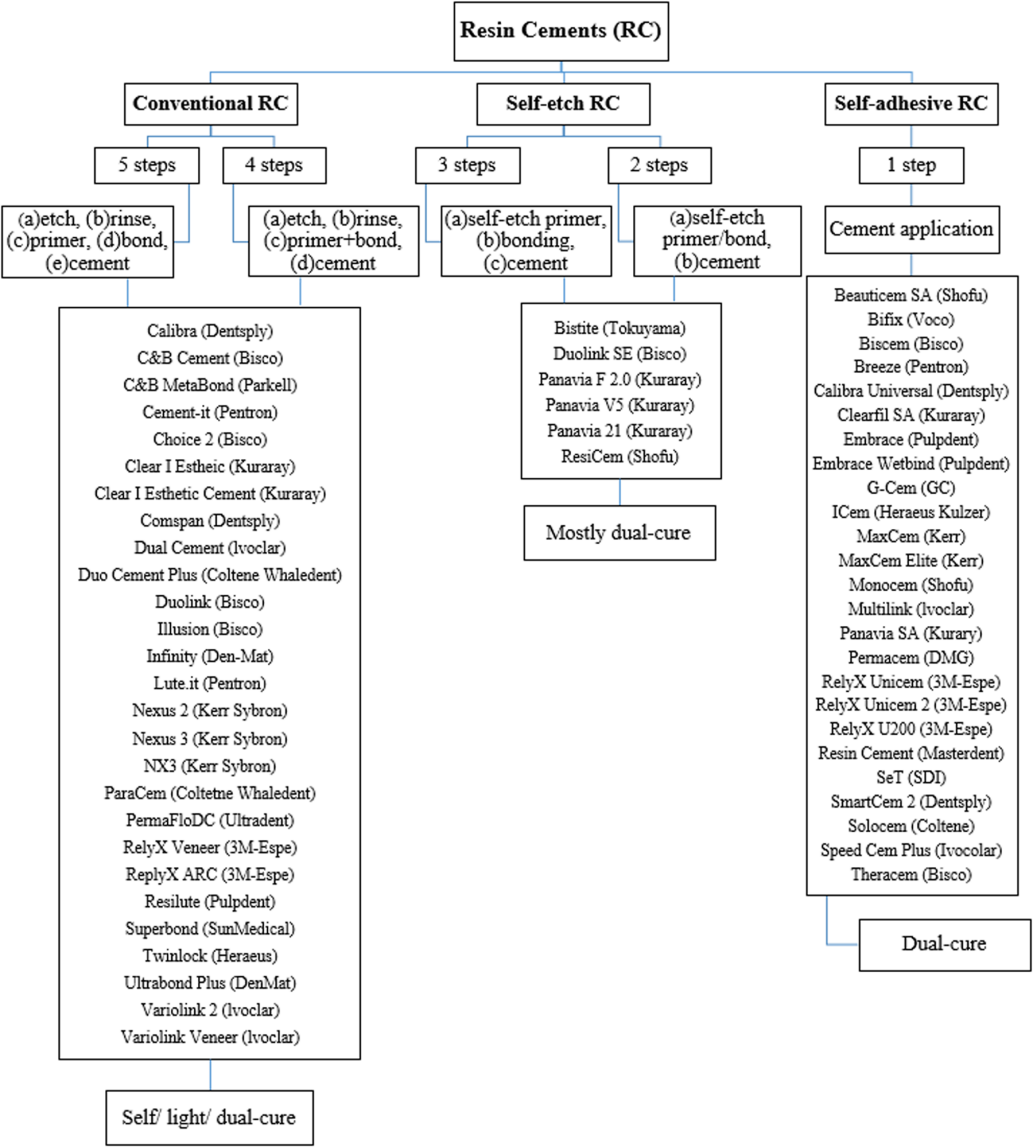
The classification of commercially available resin cements based on the application procedure.

Available resin cements could be classified as conventional (etch and rinse), self‐etch, and self‐adhesive (all‐in‐one) resin cements based on the application protocols (Figure 1) (Migliau, 2017; Pegoraro et al., 2007). Generally, resin cement selection is affected by required retention, isolation possibility, esthetic criteria, mechanical properties of restorative materials, and the bonding substrate (dentin or enamel) (Manso et al., 2011; Sunico‐Segarra & Segarra, 2015). For enamel, adhesion mainly occurs through the penetration of resin into microporosities created by acid etching (Van Landuyt et al., 2007). In the dentin, adhesion is more complex and happens when the resin entangles the exposed collagen fibers (Van Landuyt et al., 2007). Dentin porosity, hydrophilicity, and the hydroxyapatite composition of the collagen matrix compromise the adhesion in dentin (Migliau, 2017). Cementum, in comparison, is less hard and more permeable to a variety of materials (Kaneshiro et al., 2008).

The clinical outcomes, performance, and success of PCRs are largely affected by appropriate cement selection. Adhesive cements comprise a wide range of types, compositions, and characteristics (Figure 1 and Table 1) (Abo‐Hamar et al., 2005; Ashy & Marghalani, 2022; Behr et al., 2009; Borges et al., 2008; Bouillaguet et al., 2000; Burgess et al., 2010; Carvalho et al., 2004; Casselli & Martins, 2006; Cekic et al., 2007; Christensen, 2002, 2007; D'Arcangelo et al., 2009; D'Arcangelo, De Angelis, Vadini, Carluccio, 2012; D'Arcangelo, De Angelis, Vadini, & D'Amario, 2012; D'Arcangelo et al., 2014; Frankenberger et al., 2008; Gregor et al., 2014; Hackman et al., 2002; Heboyan et al., 2023; Hekimoğlu et al., 2000; Kilinc et al., 2011; Manso & Carvalho, 2017; Manso et al., 2011; Meerbeek et al., 2005; Pan et al., 2015; Pegoraro et al., 2007; Petrie et al., 2001; Pissaia et al., 2015; Piwowarczyk et al., 2012; Rosenstiel et al., 1998; Sadan et al., 2005; Sensat et al., 2002; Simon & Darnell, 2012; Simon & de Rijk, 2006; Sunico‐Segarra & Segarra, 2015; Swift & Bayne, 1997; Tanoue et al., 2003; Vargas et al., 2011; Viotti et al., 2009; Vrochari et al., 2009). This review aimed at selecting resin cements for different types of PCRs and investigated if the type of restorations or restorative materials had any effect on the type of selected resin cement. The null hypothesis was that type and material have no effect on the type of resin cement selected for the cementation of PCRs.

**Table 1:** Features of different resin cement systems: advantages, disadvantages, and indications.

		Advantages	Disadvantages	Indication
Based on polymerization	Self‐cure	− No need to consider light penetration for the restoration (Simon & Darnell, 2012).	− Low color stability (Heboyan et al., 2023; Sunico‐Segarra & Segarra, 2015). − Limited available shades (Vrochari et al., 2009).	− Metal restorations and PFM (Pegoraro et al., 2007; Simon & Darnell, 2012). − Thick (more than 2 mm) ceramic restorations (Heboyan et al., 2023; Simon & Darnell, 2012). − Opaque (no glass) ceramic materials (Vrochari et al., 2009).
	Light‐cure	− Longer working time (D'Arcangelo, De Angelis, Vadini, & D'Amario, 2012; Simon & Darnell, 2012) and more color stability (D'Arcangelo, De Angelis, Vadini, & D'Amario, 2012; Pissaia et al., 2015) compared to self‐curing and dual‐curing resin cements. − Wear resistance (Hekimoğlu et al., 2000; Pissaia et al., 2015).	− Dependency on the thickness and opacity of restoration and degree of light penetration (Hackman et al., 2002; Tanoue et al., 2003).	− Low‐thickness (<2 mm), nonopaque (glass‐containing), metal‐free restorations (Borges et al., 2008; Vargas et al., 2011). − Ceramic veneer with less than 1.5 mm thickness (Hackman et al., 2002; Hekimoğlu et al., 2000; Pissaia et al., 2015; Tanoue et al., 2003).
	Dual‐cure	− High tensile and bond strength (Kilinc et al., 2011; Petrie et al., 2001; Rosenstiel et al., 1998). − High esthetic properties (Kilinc et al., 2011; Rosenstiel et al., 1998). − Radiopacity (Kilinc et al., 2011; Rosenstiel et al., 1998). − Increased durability (Kilinc et al., 2011; Rosenstiel et al., 1998).	− Difficult to handle (D'Arcangelo, De Angelis, Vadini, Carluccio, 2012). − Restricted working time (D'Arcangelo et al., 2014). − Low color stability (Hekimoğlu et al., 2000; Sunico‐Segarra & Segarra, 2015). − High retentive strength (Pan et al., 2015).	− Opaque ceramic materials (Hekimoğlu et al., 2000; Pegoraro et al., 2007; Sadan et al., 2005). − Indirect endocrowns (Gregor et al., 2014). − Metal free restorations (Simon & Darnell, 2012).
Based on generations	Conventional cements (etch and rinse)	− Highest bond strengths to enamel (Simon & de Rijk, 2006; Sunico‐Segarra & Segarra, 2015) and better bonding to indirect composite (D'arcangelo et al., 2009; Viotti et al., 2009) compared to self‐etch or self‐adhesive resin cements. − Adequate bond strengths to dentin (Casselli & Martins, 2006). − Comes in different shades. − Reduced microleakage (Swift & Bayne, 1997).	− Technique sensitive (Burgess et al., 2010; Meerbeek et al., 2005). − More steps are required compared to other types (Burgess et al., 2010). − Possibility of postoperative sensitivity of the tooth (Christensen, 2002, 2007). − Difficulty in obtaining a hermetic seal (Bouillaguet et al., 2000).	− Where the predominant remained tooth structure is enamel (Frankenberger et al., 2008; Simon & de Rijk, 2006; Sunico‐Segarra & Segarra, 2015) or highly calcified tooth structures (Sunico‐Segarra & Segarra, 2015). − Low‐strength ceramic materials (Frankenberger et al., 2008; Sunico‐Segarra & Segarra, 2015). − Enamel margins of inlays and onlays (Frankenberger et al., 2008; Sunico‐Segarra & Segarra, 2015). − Ceramic Maryland restorations (Sunico‐Segarra & Segarra, 2015).
	Self‐etch cements	− Higher bond strengths to dentin compared to conventional group (Gregor et al., 2014; Sunico‐Segarra & Segarra, 2015) while fewer steps are required (Cekic et al., 2007; Christensen, 2007). − Low technique sensitivity (Christensen, 2007). − Low postoperative sensitivity (Sensat et al., 2002; Sunico‐Segarra & Segarra, 2015). − More durability than self‐adhesive system (Sunico‐Segarra & Segarra, 2015).	− Lower bond strength to enamel than conventional group (Cekic et al., 2007; Sunico‐Segarra & Segarra, 2015). − Less shades are available compared to conventional group (Sunico‐Segarra & Segarra, 2015). − Recommended to be stored in refrigeration away from sunlight (Sunico‐Segarra & Segarra, 2015).	− Crowns and bridges where the predominant remaining structure is healthy dentin (Sunico‐Segarra & Segarra, 2015). − Compromised retention (Sunico‐Segarra & Segarra, 2015). − Inlays and onlays, particularly for teeth with large defects (Sunico‐Segarra & Segarra, 2015).
	Self‐adhesive cements Single step	− Lower technique sensitivity and fewer steps required compared to two other groups (Behr et al., 2009; Manso et al., 2011). − Low pulp irritation (Piwowarczyk et al., 2012). − Low solubility (Piwowarczyk et al., 2012) − No pretreatment is required (Carvalho et al., 2004).	− Lower bond strength to enamel compared with dentine compared to two other groups (Abo‐Hamar et al., 2005; Gregor et al., 2014; Manso et al., 2011). − Recommended to be stored in refrigeration and kept away from sunlight (Sunico‐Segarra & Segarra, 2015). − Not indicated for veneers (Manso et al., 2011).	− Where the isolation is difficult (Kaneshiro et al., 2008; Manso et al., 2011). − High‐strength (no glass) ceramics (Abo‐Hamar et al., 2005; Manso et al., 2011) − Metal‐based restorations (Abo‐Hamar et al., 2005; Manso et al., 2011) − Compromised retention (Sunico‐Segarra & Segarra, 2015) − Inlays and onlays when there are little tooth structure (Ashy & Marghalani, 2022; Sunico‐Segarra & Segarra, 2015). − When the preparation walls are mainly dentin without enamel (Abo‐Hamar et al., 2005; Manso et al., 2011).

Abbreviation: PFM, porcelain fused to metal.

## 2 MATERIALS AND METHODS

This systematic review was conducted according to the preferred reporting items for systematic reviews and meta‐analyses (PRISMA) (Liberati et al., 2009). An electronic search was performed in PubMed, Medline, Scopus, and Google Scholar databases, applying related keywords in different combinations in title, abstract, or keywords (1991–2023) (Table 2). The review was developed up to May 25, 2023. The inclusion criteria for selecting the articles were the evaluation of the clinical or experimental performance of resin cements, comparison between different resin cements, and evaluating the effect of restorative materials or surface treatments on final results in PCRs cementation. The included study types were randomized clinical trials (RCTs), review articles, and experimental studies; nonpeer‐reviewed studies were excluded, as well as animal studies, case studies or reports, clinical studies with less than 2 years of follow‐up, or articles published on patient questionnaires or interviews. Studies on other types of restorations (not PCRs) and those without any evaluation of cement performance were also excluded. Using reference management software (Endnote X9; Thomson Reuters), duplicated studies were eliminated, and articles were selected based on title–abstract and full‐text analyses by three independent authors (M. S., M. M. A., and S. A.). In cases of disagreement, the opinion of the fourth author (S. G.) was considered for decision‐making.

**Table 2:** Systematic review search strategy.

Searching strategy	(Conservative restorative treatment OR conservative prosthetic treatment OR partial coverage restoration OR inlay OR onlay OR dental veneer OR dental laminate OR Maryland bridge OR fixed partial denture OR resin bonded fixed partial denture OR resin bonded bridge OR adhesively‐retained fixed partial denture OR adhesively‐retained fixed dental prosthesis OR endocrown OR partial coverage) AND (adhesive retention OR bonding retention OR bonding ability OR dental cement OR dental adhesive OR resin cement OR luting cement OR adhesive cement OR cement*).

**Figure 2:**
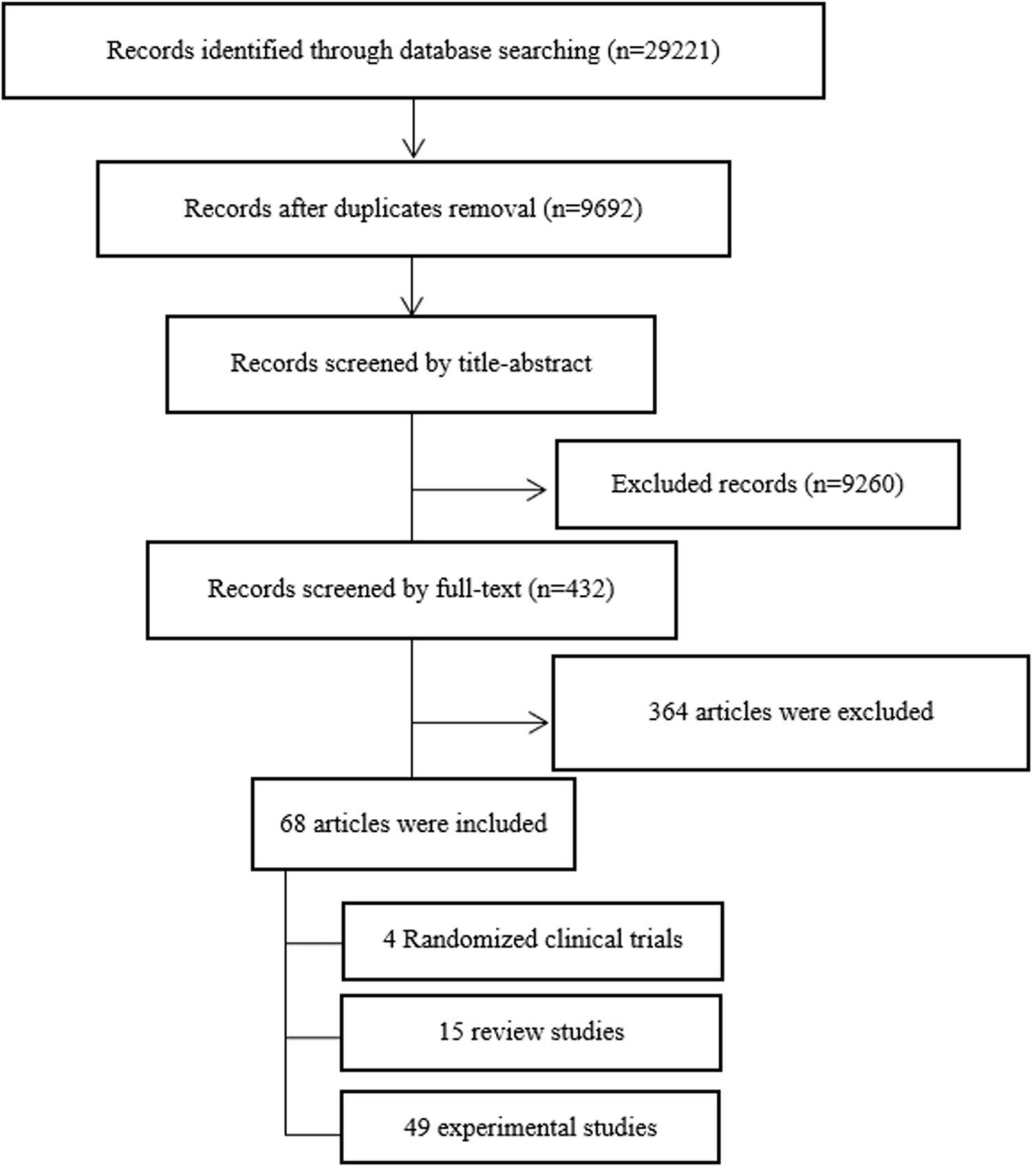
Search results flowchart diagram according to preferred reporting items for systematic reviews and meta‐analyses.

The PICOT was defined as: Population: partial coverage restorations, Intervention: cementation, Comparisons: experimental or clinical performance, Outcomes: retention and durability, Types of publications: RCTs, literature reviews, and experimental studies. The following data were extracted from each study: study design, objective, material and method used for PCRs cementation, type of cement used for the restoration, and comparison made between different cements. The number of search results for the selected keywords was 4904 in PubMed, 18,400 in Google Scholar, and 5917 in Scopus. After removing duplicates, 9692 records remained. After title–abstract analysis, 432 studies were selected for full‐text review. Finally, 68 studies met the requirements for inclusion and exclusion criteria (Figure 2).

## 3 RESULTS

The advantages, disadvantages, and indications of resin cements were among the interesting subjects in the literature. There were several studies (14 articles) on the characteristics of self‐etch and self‐adhesive cements (single step); however, fewer studies (five articles) were available on their indications (Table 1). However, Manso et al. (2011) and Abo‐Abo‐Hamar et al. (2005) had extensively dealt with the indications of these cements. There was no article summarizing the classification of different types of resin cements and commercial brands in the studies reviewed. Therefore, the present study also dealt with the appropriate product for cementation among the available resin cements based on the literature reviewed (Figure 1). In addition, 12 catalogs were also consulted to compile a table that summarized the manufacturer's recommendations on the use of resin cements in various situations (Table 3) (3M Dental Products Laboratory, 1998; 3M Oral Care, 2016; Dentsply Sirona, 2016, 2017; GC America Inc., 2023; Inside Dentistry, 2019; Ivoclar Vivadent AG, 2018a, 2018b; Kuraray Noritake Dental Inc., 2018; PANAVIA 21 brochure, (n.d.); PANAVIA F 2.0 brochure, 2012; Panavia SA Cement Universal, 2019).

**Table 3:** Manufacturers' recommendations for selecting proper resin cement.

Manufacturer cements		Classification		Indication
Bisco, Inc. (Inside Dentistry, 2019)	Choice 2	Light‐cure/conventional		Feldspathic/pressed veneer, lithium disilicate veneer, composite reinforced veneer.
	Theracem	Self‐adhesive		Feldspathic/pressed inlay and onlay, lithium disilicate inlay and onlay, composite reinforced inlay and onlay.
	Duo‐Link Universal	Self‐adhesive		Feldspathic/pressed inlay and onlay, lithium disilicate inlay and onlay, composite reinforced inlay and onlay, metal/PFM Maryland bridge.
	eCEMENT L/C	Light‐cure		Feldspathic/pressed veneer, lithium disilicate veneer.
	eCEMENT D/C	Dual‐cure		Lithium disilicate inlay and onlay.
3M ESPE	RelyX Veneer (3M Oral Care, 2016)	Light‐cure/conventional		Glass ceramic veneer, resin composite veneer, resin nanoceramic veneer.
	RelyX ARC (3M Dental Products Laboratory, 1998)	Dual‐cure/conventional		Metal/PFM inlay and onlay (secondary recommendation)/Maryland bridge, porcelain/ceramic/composite inlay and onlay.
	RelyX Unicem/RelyX Unicem 2 (3M Oral Care, 2016)	Self‐adhesive		Metal/metal based inlay and onlay/Maryland bridge, glass ceramic inlay and onlay, oxide ceramic inlay and onlay/Maryland bridge, resin composite inlay and onlay.
	RelyX Ultimate (3M Oral Care, 2016)	Combination of conventional and self‐etch		Metal/metal based inlay and onlay/Maryland bridge, glass ceramic inlay and onlay/veneer, oxide ceramic inlay and onlay/Maryland bridge, resin composite inlay and onlay/veneer, resin nanoceramic inlay and onlay/veneer.
Ivoclar Vivadent	Variolink 2 (Ivoclar Vivadent AG, 2018b)	Dual‐cure/conventional		Metal‐free restorations: veneers/inlays and onlays, IPS e.max glass‐ceramic restorations, IPS empress restorations, composite restorations.
	Variolink Esthetic (Ivoclar Vivadent AG, 2018a)	Light‐cure		Glass‐ceramics veneers/inlays and onlays/partial crowns, lithium disilicate veneers/occlusal veneers/inlays and onlays/partial crowns, hybrid ceramics and composites veneers/inlays and onlays.
		Light/dual‐cure		Same as above, metal/metal‐based Maryland bridges.
	SpeedCEM Plus (Ivoclar Vivadent AG, 2018a)	Self‐adhesive		Metal/metal‐based inlays and onlays/partial crowns.
Dentsply Sirona	Calibra Ceram (Dentsply Sirona, 2017)	Light‐cure		Veneer.
	Calibra Veneer (Dentsply Sirona, 2017)	Adhesive cement		Inlay and onlay.
	Calibra Universal (Dentsply Sirona, 2016)	Self‐adhesive	Self‐cure	Metal, PFM, resin/composite, ceramic and porcelain inlays, onlays, crowns and bridges and endodontic posts.
			Light‐cure	Translucent ceramics and composites.
			Dual‐cure	PFM, zirconia, alumina, opaque ceramics, and composites.
GC America	G‐CEM (GC America Inc., 2023)	Self‐adhesive		Metal/PFM/ceramics—low and middle strength/composite/reinforced polymer inlay and onlay.
	G‐CEM LinkForce (GC America Inc., 2023)	Self‐adhesive		Metal/PFM/ceramics—low and middle strength/composite/reinforced polymer inlay and onlay, veneers.
	G‐CEM ONE (GC America Inc., 2023)	Self‐adhesive		Same as above.
Kuraray Noritake		Self‐etch	Tooth color	Posterior adhesion bridge and splint, metal inlay and onlay, silanated porcelain or cured composite inlay and onlay.
			Standard white	Posterior adhesion bridge and splint, metal inlay and onlay.
			Opaque	Anterior adhesion and splint, posterior adhesion bridge and splint.
	Panavia F 2.0 (PANAVIA F 2.0 brochure, 2012)	Dual‐cure/self‐etch		Metal/metal alloys (e.g., gold or titanium)/metal oxide ceramics/silica‐based ceramics/Hybrid ceramics/composites veneer/inlay and onlay.
	Panavia V5 (Kuraray Noritake Dental Inc., 2018)	Self‐etch		Veneer.
	Panavia SA (Panavia SA Cement Universal, 2019)	Self‐adhesive		Inlay and onlay.

Abbreviation: PFM, porcelain fused to metal.

Improvements in esthetic restorative materials and technologies have caused an ever‐increasing application of PCRs in routine dental practices. Since dealing with the cementation of PCRs calls for familiarity with these restorations, different types of PCRs will be briefly elaborated, and then the resin cement selection criteria will be discussed based on the articles reviewed.

### 3.1 Inlay, onlay, and vonlay

Inlay is a minimally invasive restoration that restores small to medium dental lesions, (Felden et al., 1998) onlay is used for medium to extensive defects with cuspal coverage, (Felden et al., 1998; Ferro et al., 2017) and vonlay is a combination of onlay and ceramic veneer (McLaren et al., 2015). These PCRs can be made of metal, ceramic (preferably), or composite materials (McGill & Holmes, 2012). Ceramics have more surface hardness, wear resistance, (Mörmann et al., 2013) biocompatibility, (St. John, 2007) and acceptable resistance to compressive loads (Fron Chabouis et al., 2013). However, structural brittleness, (Ansong et al., 2013) susceptibility to shear stresses, the wear of opposing tooth structure, and time‐consuming and costly manufacturing are among the drawbacks (Fron Chabouis et al., 2013; Mörmann et al., 2013). Composite PCRs are less costly, time‐saving, and have acceptable wear resistance (Chang & Kim, 2014; Fron Chabouis et al., 2013). However, disadvantages like less fracture resistance, (Costa et al., 2014) polymerization shrinkage, marginal microleakage, and toxicity resulting from incomplete polymerization (Darmani et al., 2007; Durner et al., 2010) have been reported.

### 3.2 Occlusal veneer and overlay

Occlusal veneer is an ultrathin, bonded treatment for teeth that are worn down or eroded in the occlusal surface, (Magne et al., 2010) and overlay covers all the cusps (Felden et al., 1998) for correcting the anatomy of posterior teeth. They have many advantages, such as protection of dental structure, recovery of masticatory function and esthetic, (Schlichting et al., 2016; Yazigi et al., 2017) simplicity of cementation, (Carvalho et al., 2014) and being more conservative than onlays and full‐coverage crowns (Magne et al., 2010; Schlichting et al., 2016).

### 3.3 Endocrown

Endocrown is a single PCR made of acid‐etchable metal ceramic, ceramic, or composite materials for endodontically treated teeth with large coronal destruction (Biacchi & Basting, 2012). This monoblock restoration is an alternative to post and crown with many advantages, such as an easy and time‐saving preparation procedure, esthetic appeal, resistance to failure, and conservation of tooth structure (Biacchi et al., 2013; Dietschi et al., 2008). Endocrown relies on macro‐ and micromechanical retention (Biacchi & Basting, 2012; Biacchi et al., 2013).

### 3.4 Laminate veneer

Laminate veneer is a thin indirect ceramic or composite layer bonded to the tooth's facial surface (da Cunha et al., 2014) to reproduce a natural appearance with minimal or no preparation (da Cunha et al., 2014; Layton & Walton, 2007). Dental laminate is indicated to restore discolored, malpositioned, or malcontoured teeth (Alabdulwahhab et al., 2015). Clinical studies have reported good results for more than 10 years (Layton & Walton, 2007; Layton et al., 2012).

The PCR could also be used as a retainer for fixed prostheses to reduce the extension of tooth preparation. This type of prosthesis uses metallic or ceramic extensions bonded to adjacent teeth and could be a Maryland bridge, laminate bridge, or inlay bridge (Edelhoff et al., 2016; Pahlevan, 2006; Trushkowsky, 2008). These types of PCR‐retained prostheses have the same considerations for bonding as single‐tooth PCRs; however, the bonding, loading, and occlusal considerations should be followed more precisely. Considering the higher failure rate, PCR‐retained prostheses could be indicated for temporary restorations, small spans, and younger patients with lower bite force (Ibbetson, 2004; Trushkowsky, 2008). These restorations, generally, are not suggested in deep vertical overlap, long‐span edentulous space, when the abutments are mobile, or when the patient has parafunctional habits (Ibbetson, 2004).

## 4 DISCUSSION

PCR is an indirect fixed prosthesis that tries to replace the demolished tooth structure while preserving more remaining tooth structure compared to conventional prostheses (Donovan & Chee, 1993). This type of restoration aims to recover full mechanical function, strength, and esthetic while protecting the remaining tooth structure, improving periodontal health through the accessibility of margins, simplifying daily maintenance, and reducing gingival and pulpal irritations (Dallı et al., 2012; Ruiz, 2015). Selecting the appropriate resin cement is one of the key factors that determines the success and longevity of PCR (Santos et al., 2009). Cement in this restoration provides not only retention but also stability, appearance, and durability. The studied articles demonstrated that the material type, design, thickness, and opacity of PCR affect the cement type selection as well as the applied load and dental substrate to be bonded (Borges et al., 2008; Hackman et al., 2002; Hekimoğlu et al., 2000; Tanoue et al., 2003). This highlighted the rejection of the null hypothesis. In the next paragraphs, the results of the reviewed articles are summarized based on the queries a dentist might ask in a clinical situation for cementing a PCR with resin cement:

### 4.1 Light, dual, or self‐cure resin cement?

Light‐cure resin cements are not suitable for metallic restorations; however, they could be, and in fact, they are preferred to be used for metal‐free PCRs (Borges et al., 2008; Vargas et al., 2011). Ceramic PCRs are relatively thin, and their appearance is affected by the cement color; light‐cured resin cements are generally preferred to provide immediate final polymerization, esthetic, and strength. Light‐cure cements offer sufficient working time, facilitate excess cement removal before polymerization, provide better color stability as they do not contain chemical amine initiators, cure completely in a shorter time, and quickly seal the margins compared to self‐ and dual‐cured resins (Simon & Darnell, 2012; Simon & de Rijk, 2006; Tanoue et al., 2003). However, for areas with difficult access or where the curing light cannot penetrate (due to opacity, thickness, or material type), self‐ or dual‐cure resin cements could be used (Heboyan et al., 2023; Simon & Darnell, 2012). Dual‐cure cement can be set through chemical reaction alone; however, light curing is necessary to reach the maximum degree of polymerization (El‐Badrawy & El‐Mowafy, 1995; Manso et al., 2011). When the thickness of material is more than 1.5 mm in low‐glass ceramics (e.g., lithium disilicate, zirconia lithium silicate, and glass‐infiltrated ceramics), dual‐cure resin cements are indicated, while translucent ceramics (feldspathic or leucite‐reinforced ceramics) could be cemented reliably by light‐curable resin cements (Borges et al., 2008; Hackman et al., 2002; Simon & Darnell, 2012).

### 4.2 Which type of resin cement is preferred?

Conventional resin cement (etch and rinse type) provides predictable bond strength to enamel with proven long‐term clinical success (Peumans et al., 2005; Simon & de Rijk, 2006; Swift & Bayne, 1997). The bonding mechanism to dentin is through resin penetration in exposed collagen fibrils (Peumans et al., 2005). This penetration could provide high bond strength if the steps are followed properly; however, multiple steps in conventional resin cements and the effect of the water content of dentin might compromise the efficiency of dentin bonding (Burgess et al., 2010; Casselli & Martins, 2006). Self‐etch resin cement, although having a weaker bond to enamel, provides higher bond strength in dentin (Cekic et al., 2007; Simon & de Rijk, 2006). Self‐etch systems (with a pH of about 2) could not expose the collagen fibers completely for acceptable cement penetration, and additional ionic bonding and specific functional monomers are needed to enhance their adhesive efficiency (Van Landuyt et al., 2007; Peumans et al., 2005). Functional monomers are classified based on their bonding potential; 10‐methacryloyloxydecyl dihydrogen phosphate (10‐MDP), for instance, could establish a strong and stable chemical bond with hydroxyapatite, which increases the diffusion and adhesion of self‐etch resin cement (Ashy & Marghalani, 2022; Carvalho et al., 2004; Wang et al., 1991; Watanabe et al., 1994). Self‐adhesive cement does not require dentin conditioning (Tay et al., 1995). Although the bond strength of self‐adhesive resin cements to dentin and enamel has been reported to be adequate, it is significantly less than conventional or self‐etch types (Carvalho et al., 2004; Simon & de Rijk, 2006). Self‐etching and self‐adhesive cements are particularly indicated for teeth with extensive defects when the predominant exposed structure is dentin. However, even in such a situation, self‐etching cement is preferred because of its higher and more durable bond strength (Manso et al., 2011; Sunico‐Segarra & Segarra, 2015).

### 4.3 Does restorative material have any effect?

PCR may be fabricated from metal or esthetic nonmetallic materials (different ceramics and composite resins) (Peutzfeldt et al., 2011). The ceramic materials could be divided into etchable glass ceramic (silica‐based) and nonetchable no‐glass (e.g., zirconia) ceramic subgroups (Peutzfeldt et al., 2011; Sunico‐Segarra & Segarra, 2015). For the cementation of metal PCRs, self‐cure cements are highly recommended (see Table 1 for details) and could be used in conventional cementation or adhesive luting modes (Pegoraro et al., 2007; Simon & Darnell, 2012). There is no way for light to penetrate through metal; however, considering the probability of light penetration through tooth structure, a dual‐cure cement might still be efficient with a high degree of conversion and good physical properties (Manso et al., 2011). Some resin cement manufacturers suggest special types of their products for each type of PCR (Table 3) (3M Dental Products Laboratory, 1998; 3M Oral Care, 2016; Dentsply Sirona, 2016, 2017; GC America Inc., 2023; Kuraray Noritake Dental Inc., 2018; Inside Dentistry, 2019; Ivoclar Vivadent AG, 2018a, 2018b; PANAVIA 21 brochure, (n.d.); PANAVIA F 2.0 brochure, 2012; Panavia SA Cement Universal, 2019). As an example, a dual‐cure adhesive resin cement (RelyX Ultimate, 3M‐Espe) has been recommended for metal inlays and onlays, (3M Oral Care, 2016) or a self‐adhesive resin cement (SpeedCem Plus; Ivoclar Vivadent) has been suggested for metal‐based PCRs by the manufacturer (Ivoclar Vivadent AG, 2018a). Ceramic restorations could affect light penetration because of their thickness and opacity. Thick (above 1.5–2 mm) and opaque (no‐glass) ceramics inhibit light penetration, and therefore, self‐ or dual‐cure cements could provide more predictable results (Hekimoğlu et al., 2000; Sadan et al., 2005; Simon & Darnell, 2012). Thin (<1.5 mm) or more translucent materials (high glass ceramic), however, could benefit from the advantages of light‐curable, conventional resin cements (Borges et al., 2008; Vargas et al., 2011). Inlays, onlays, laminate veneers, and other PCRs fabricated from high‐glass ceramics or composites could take advantage of adhesive cementation with light‐curable cements in total‐etch mode (Borges et al., 2008; Hekimoğlu et al., 2000; Pissaia et al., 2015; Simon & de Rijk, 2006; Vargas et al., 2011). Dual‐cure resin cements could also be recommended considering their color and opacity varieties, low solubility in oral fluids, high radiopacity, high bond strength to dental tissues, and increased durability (Kilinc et al., 2011; Pegoraro et al., 2007; Rosenstiel et al., 1998). In a 5‐year prospective clinical evaluation, total etch dual‐cure resin cement showed better clinical performance regarding marginal discoloration and marginal adaptation than self‐etch and self‐adhesive resin cements (Eltoukhy et al., 2021). Time‐dependent discoloration attributed to tertiary amine content in dual‐cure cements, (Kilinc et al., 2011; Rosenstiel et al., 1998) however, calls for the application of amine‐free versions. Variolink Esthetic DC (Ivoclar Vivadent), Panavia V5 (Kuraray Noritake Dental), NX3 Nexus (Kerr Dental), and G‐CEM Linkforce (GC Corporation) are among amine‐free dual cure resin cements introduced for use in esthetic veneers when light cure cement could not be applied (Atay et al., 2019).

### 4.4 How to prepare the surface of the restoration?

Adhesion with resin cement calls for surface preparation of restorative materials to provide stable and durable adhesive bonding (Stewart et al., 2002). Table 4 summarizes the available studies comparing the effect of different surface preparations on the bond strength of resin cements to different surfaces (Casucci et al., 2011; Chatterjee & Ghosh, 2022; D'Arcangelo et al., 2009; Duarte et al., 2008; Hitz et al., 2012; Murthy et al., 2014; Özdemir et al., 2019; Pisani‐Proenca et al., 2006; Raeisosadat et al., 2020; Shimada, 2002; Turp et al., 2016; Upadhyaya et al., 2019).

**Table 4:** Studies' outcomes on different resin cements and surface pretreatments.

References	Material type	Comparison	Conclusions
Shimada (2002)	Glass ceramic	Microshear bond strength of dual‐cured resin cement with different pretreatments: sandblasting, etching, and silanization.	Silane coupling agent + acidic primer could significantly increase the bond strength of castable glass ceramic (Olympas) to cement.
Pisani‐Proenca et al. (2006)	Lithium disilicate (LDS) glass ceramic	The microtensile bond strength of three resin cements (self‐adhesive: RelyX Unicem, resin‐based luting agents: Multilink and Panavia F) to ceramic submitted to two surface treatments (no conditioning, or hydrofluoric acid + silane).	Etching and silanization treatments significantly increased resin bonding to LDS ceramic, regardless of the resin cement used.
Duarte et al. (2008)	Composite resin	The effect of acid pretreatment on microtensile bond strengths of self‐adhesive and self‐etch resin cements to enamel.	Etching with phosphoric acid significantly increased bond strengths in self‐adhesive cement but did not improve the bond strengths in self‐etching cement.
D'Arcangelo et al. (2009)	Composite resin and leucite‐based glass ceramic	Microtensile bond strength of resin cements (conventional, self‐etch, and self‐adhesive) for dentin bonding.	Conventional resin cements provided more reliable bonding for indirect resin‐based composite restorations. In contrast, self‐adhesive cement showed the highest mean bond strength for glass ceramic.
Casucci et al. (2011)	Zirconia ceramic	Effect of different surface treatments (airborne particle abrasion (S), selective infiltration etching (SIE), hot etching solution (ST), and no treatment) on microtensile bond strength to resin cement.	Bond strength values were significantly higher by SIE and ST treatments compared to S and control group.
Hitz et al. (2012)	Silica‐based glass ceramic	Shear bond strengths in six self‐adhesive resin cements and a conventional resin cement to dentin.	Considering the bond strength, not all self‐adhesive resin cements could be a valid alternative to conventional resin cements.
Murthy et al. (2014)	Zirconia ceramic	Effect of different surface treatments on shear bond strength of resin cements: sandblasting with 110 μm alumina, sandblasting with 250 μm alumina, acid etching with 9.6% HF, and laser.	Laser treatment increased the shear bond strength value significantly.
Turp et al. (2016)	Zirconia ceramic	Effect of different surface treatments on microtensile bond strength of resin cements: air‐particle abrasion, air‐particle abrasion and zirconia primer, air‐particle abrasion and 10‐MDP containing primer.	10‐MDP containing resin cements and primer increased the bond strength of resin cement to zirconia.
Upadhyaya et al. (2019)	LDS	Shear bond strength of ceramic to dentin by conventional, self‑etch, and self‑adhesive resin cements.	Conventional resin cement produced significantly higher bond strength.
Özdemir et al. (2019)	Zirconia	The shear bond strength of dual‐ and self‐cure resin cements with different surface treatments (Co‐Jet; Nd:YAG laser; Er:YAG laser; Nd‐YAG laser + silane; Er‐YAG laser + silane; Co‐Jet + bonding agent; Nd:YAG laser + silane + bonding agent; Er:YAG laser + silane + bonding agent).	Co‐Jet + bonding showed the highest values, and Nd:YAG laser showed the lowest. MDP‐based silane + bonding increased the shear bond strength in each group.
			Dual‐cure cement showed significantly higher bond strength compared to self‐cure cement.
Raeisosadat et al. (2020)	Base metal alloy (nickel–chrome alloy)	Effect of different surface treatments on the shear bond strength of resin cement: sandblasting, Er:YAG laser, Er:YAG laser after sandblasting, MKZ metal primer after sandblasting.	Er:YAG laser treatment provided the highest shear bond strength between metal alloy and resin cement.
Chatterjee and Ghosh (2022)	Zirconia ceramic	Effect of different surface pretreatments (mechanical, chemical, mechanochemical) on the shear bond strength of resin cements (self‑adhesive cements, 10‑MDP containing cements, bis‑GMA cements).	The ideal zirconia preparation protocol was using a combination of sandblasting with 50 μm Al_2_O_3_ particles and self‐adhesive resin cement containing 10‑MDP.

Abbreviations: 10‐MDP, 10‐methacryloyloxydecyl dihydrogen phosphate; Er:YAG, erbium‐doped yttrium aluminum garnet; Nd:YAG, neodymium‐doped yttrium aluminum garnet.

For metal substructures, sandblasting, or using chemicals, namely metal primer, tin plating, and silica coating, have been proposed (Denizoglu et al., 2009; Parsa et al., 2003). Sandblasting (by 50‐ µm Al_2_O_3_ particles under 0.1–0.6 MPa pressure) is a low‐cost, accessible procedure that improves adhesion and surface wettability by mechanically removing debris and increasing surface roughness and porosities (Abreu et al., 2009; Al Jabbari et al., 2012; Gurbuz et al., 2008; Lahori et al., 2014). Metal primers have active components to aid in the retention of resin on the metal surface. 10‐MDP proved to increase resin cement bonding to base metal alloys (Taira et al., 2004; Watanabe et al., 2003). However, tin plating and silica coating (e.g., using Rocatec Technology) require additional equipment and are considered technique‐sensitive, which reduces their applicability in dental offices (Imbery et al., 1992; Petrie et al., 2001; Watanabe et al., 2008).

Silica‐based ceramics have shown high bonding strength (up to 71.5 MPa) to resin cement, (Kamada et al., 1998; Nagai et al., 2005; Roulet et al., 1995) provided that correct preparation methods are followed. Sandblasting with 50‐µm aluminum oxide particles (at 80 psi) or 4%–9.5% hydrofluoric acid (HF) etching followed by subsequent silanization have been proposed for surface preparation (Kamada et al., 1998; Manso et al., 2011; Roulet et al., 1995). The glass phase will be dissolved in HF to create micromechanical retention (Borges et al., 2003). Etching with HF and silane application is preferred over air abrasion since a higher failure rate and complications have been reported with the latter on thin veneers (Friedman, 1998; Shaini et al., 1997). Etching and silanization do not form a suitable bond for nonglass acid‐resistant ceramics like zirconia and alumina (Heikkinen et al., 2007; Watanabe et al., 1994). Alternative conditioning procedures include the elimination of contaminating phosphate from the zirconia bonding surface, tribochemical silica coaing, (Amaral et al., 2006; Comino‐Garayoa et al., 2021) or alumina air abrasion (by 50‐µm Al_2_O_3_ particles under 0.1–0.25 MPa pressure), (Raeisosadat et al., 2020) and the application of 10‐MDP‐containing resin cements for achieving chemical bonding (Comino‐Garayoa et al., 2021; Pisani‐Proenca et al., 2006; Wang et al., 1991). Sandblasting, (Santos et al., 2009) followed by 10‐MDP monomer application, (Atsu et al., 2006) selective infiltration etching, (Aboushelib et al., 2007) chemical etching, (Casucci et al., 2009) and laser irradiation, (Inokoshi et al., 2014) have been suggested for zirconia, while sandblasting followed by phosphoric acid etching to clean the surface (Jivraj et al., 2006) or silane application (Soares et al., 2005) have been recommended for indirect composite or resin‐based hybrid ceramic restorations. Murthy et al. (2014) studied the effect of different surface treatments on the shear bond strength of resin cements to zirconia. The surface treatments included sandblasting with 110 or 250 μm alumina at 35 psi for 15 s from a distance of about 10 mm, acid etching with 9.6% HF, and laser. The results showed a significant increase in shear bond strength after laser treatment. Shimada (2002) researched the microshear bond strength of dual‐cured resin cement with different pretreatments, namely sandblasting, etching, and silanization. The silane coupling agent and acidic primer caused a significant increase in the bond strength of castable glass ceramic (Olympas) to cement.

The tooth surface also needs preparative procedures to provide long‐lasting, predictable bonding. Etch‐and‐rinse cement proved to provide higher bond strength, especially on enamel surfaces (Simon & de Rijk, 2006; Swift & Bayne, 1997).

The present study tried to review the available studies on resin cement selection in different types of PCRs. However, the main restriction returns to the limited clinical studies on the long‐term durability of bonding provided by resin cements. Further studies on new restorative materials and the long‐term durability of bonding by improved versions of cements are encouraged, as are evaluative studies on cement durability in challenging situations, namely structural deficiencies of enamel or dentin (e.g., amelogenesis or dentinogenesis imperfecta), excessive loading situations, and reduced height or width of bonded abutments.

## 5 CONCLUSION

Based on the results of the literature review, the following conclusions can be drawn:

- Self‐ and dual‐cure resin cements have been recommended for the cementation of metallic PCRs (for conventional cementation or adhesive luting).
- The PCRs fabricated from thin (<1.5–2 mm), translucent (high‐glass), and low‐strength ceramics or composites could be adhesively bonded by light‐cure and conventional (etch and rinse) adhesive resin cements. Thick and opaque ceramic restorations could be cemented by self‐curing or dual‐curing resin cements.
- Self‐etching and self‐adhesive cements, especially those in dual‐cure types, are not indicated for laminate veneers.

## AUTHOR CONTRIBUTIONS

**Conceptualization**: Safoura Ghodsi, Sarah Arzani, Mina Shekarian, Mohammad Mostafa Aghamohs, Sasan Rasaeipour. **Data curation**: Safoura Ghodsi, Sarah Arzani, Mina Shekarian, Mohammad Mostafa Aghamohs. **Formal analysis, methodology, and project administration**: Sarah Arzani and Safoura Ghodsi. **Investigation**: Sarah Arzani and Sasan Rasaeipour. **Supervision**: Safoura Ghodsi, Sarah Arzani, and Sasan Rasaeipour.

## CONFLICT OF INTEREST STATEMENT

The authors declare no conflict of interest.

## Data Availability

Data sharing is not applicable since it was a review article and no new data were created or analyzed in this study.
